# Depletion of Key Meiotic Genes and Transcriptome-Wide Abiotic Stress Reprogramming Mark Early Preparatory Events Ahead of Apomeiotic Transition

**DOI:** 10.3389/fpls.2016.01539

**Published:** 2016-10-26

**Authors:** Jubin N. Shah, Olga Kirioukhova, Pallavi Pawar, Muhammad Tayyab, Juan L. Mateo, Amal J. Johnston

**Affiliations:** ^1^Laboratory of Germline Genetics & Evo-Devo, Centre for Organismal Studies, University of HeidelbergHeidelberg, Germany; ^2^Leibniz Institute of Plant Genetics and Crop Plant ResearchGatersleben, Germany; ^3^Centre for Organismal Studies, University of HeidelbergHeidelberg, Germany

**Keywords:** stress response, asexual reproduction, apomixis, apomeiosis, NAC transcription factors, gene regulatory network, DNA methylation, *Boechera*

## Abstract

Molecular dissection of apomixis – an asexual reproductive mode – is anticipated to solve the enigma of loss of meiotic sex, and to help fixing elite agronomic traits. The Brassicaceae genus *Boechera* comprises of both sexual and apomictic species, permitting comparative analyses of meiotic circumvention (apomeiosis) and parthenogenesis. Whereas previous studies reported local transcriptome changes during these events, it remained unclear whether global changes associated with hybridization, polyploidy and environmental adaptation that arose during evolution of *Boechera* might serve as (epi)genetic regulators of early development prior apomictic initiation. To identify these signatures during vegetative stages, we compared seedling RNA-seq transcriptomes of an obligate triploid apomict and a diploid sexual, both isolated from a drought-prone habitat. Uncovered were several genes differentially expressed between sexual and apomictic seedlings, including homologs of meiotic genes *ASYNAPTIC 1* (*ASY1*) and *MULTIPOLAR SPINDLE 1* (*MPS1*) that were down-regulated in apomicts. An intriguing class of apomict-specific deregulated genes included several NAC transcription factors, homologs of which are known to be transcriptionally reprogrammed during abiotic stress in other plants. Deregulation of both meiotic and stress-response genes during seedling stages might possibly be important in preparation for meiotic circumvention, as similar transcriptional alteration was discernible in apomeiotic floral buds too. Furthermore, we noted that the apomict showed better tolerance to osmotic stress *in vitro* than the sexual, in conjunction with significant upregulation of a subset of NAC genes. In support of the current model that DNA methylation epigenetically regulates stress, ploidy, hybridization and apomixis, we noted that *ASY1, MPS1* and *NAC019* homologs were deregulated in *Boechera* seedlings upon DNA demethylation, and *ASY1* in particular seems to be repressed by global DNA methylation exclusively in the apomicts. Variability in stress and transcriptional response in a diploid apomict, which is geographically distinct from the triploid apomict, pinpoints both common and independent features of apomixis evolution. Our study provides a molecular frame-work to investigate how the adaptive traits associated with the evolutionary history of apomicts co-adapted with meiotic gene deregulation at early developmental stage, in order to predate meiotic recombination, which otherwise is thought to be favorable in stress and low-fitness conditions.

## Introduction

Sexual reproduction, otherwise known as amphimixis, is the most prevalent mode of reproduction found across phylogenetic boundaries of multi-cellular organisms over two billion years. Sex leads to genetic recombination, allowing natural selection to act upon individual traits and segregation of mutations from the natural population (Smith, 1971). The evolutionary advantages of meiotic recombination have long been debated in biology. The deterministic mutation hypothesis sees recombination as a mean to efficiently purge deleterious mutations (Kondrashov, 1988). The Red Queen hypothesis favors recombination as an optimal way of adaptation in arms races with parasites (Jaenike, 1978). Fisher suggested that recombination might accelerate species adaptation to a changing environment by bringing beneficial allele combinations together (Fisher, 1930). Whereas Fisher’s proposal is fully supported by a recent molecular experiment in yeast (McDonald et al., 2016), it was also extensively debated by others. For instance, there are cases where recombination breaks fitter-combinations more than it does so to create novel beneficial mutation sets (Eshel and Feldman, 1970). A compromising “fitness-associated recombination (FAR)” theory proposes increase in recombination as a consequence of stress-associated DNA damage in individuals with lower fitness thus less tolerant to stress, in stark contrast to better adapted organisms bearing beneficial genetic combinations. Therefore, FAR would favor preserving the fitter genotypes, and enhance segregation of alleles in progenies of the less fit ones thus enabling new genetic combinations to occur and to be exposed to selective pressure (Hadany and Beker, 2003a,b). Following the “restoration” view of Bernstein and co-workers that sex evolved for the repair of DNA lesions, a refined hypothesis proposes that meiosis has selectively been retained in evolution mainly for its role in repair of DNA damage caused by oxidative stress and reactive oxygen species (ROS), instead of its role in generation of recombination [summarized in (Horandl and Hadacek, 2013)]. The latter view advocates that redox status between oxidized DNA and a key meiotic protein could be necessary for generating double-strand breaks (DSB), and could possibly be considered as a consequence of genome-wide epimutations caused by DNA or histone methylation changes that restrict deleterious transposable elements (de Massy, 2013; Zamudio et al., 2015). Together, the paradoxical view of sex in biology has seen several changes over the past decades, and the most recent ones address stress, fitness, DNA repair, epigenetics and *trans*-generational responses as key underlying determinants.

Asexual reproduction, on the other end, represents offspring propagation from somatic cells, including deviation of the sexual pathway by avoidance of meiotic recombination. The latter has been infrequently observed in some Eukaryotes, and is considered to be disadvantageous in a population in the long run impeding adaptation, since deleterious mutations may accumulate in the absence of meiotic recombination due to Muller’s Ratchet (Muller, 1964; Charlesworth, 2006), as experimentally documented in yeast (McDonald et al., 2016). Despite the popular views of Fisher (1930) and Muller (1964), the dynamics of adaptation in asexual populations has constantly been debated. For instance, ‘clonal interference’ theory postulates that many single beneficial mutations of varying effect contend for fixation in a two-loci model in asexuals (Gerrish and Lenski, 1998). Kim and Orr (2005) could not see that sex could be advantageous over asexuality, when multiple beneficial mutations are involved in a finite population. Multiple-mutations model of (Desai et al., 2007) assumes that all clonal mutations are of the same beneficial effect and that clonal competition between clones bearing different numbers of beneficial mutations might occur among genotypes. The problem here is that superior clonal competitors might not emerge under the assumption that beneficial mutations are of the same effect. An addendum to the latter (Sniegowski and Gerrish, 2010) discussed that asexual populations might harbor mutations of different effects, and that competitions might arise between clones that bear multiple beneficial mutations. Adaptation rate in these populations should, however, assess both beneficial and deleterious mutations, population bottlenecks, and clonal interference (Campos and Wahl, 2010). Summarizing the old and new population theories, evolution of asexuality could often be associated with genetic conflicts deviating from a faithful sexual meiotic program, and asexuals could suffer from reduced fitness and an increase in genetic drift, but the population size and the number of mutations and their interactions might play a lead role in deciding whether or not clonal populations are advantageous.

Asexual organisms do not switch ploidy cycles between generations, and asexuality is predominantly found in polyploid organisms; therefore, asexuality and polyploidy seem to be positively correlated. Meiosis fails to a large extent in polyploids, specifically in organisms with odd numbered genomes, potentially due to difficulty in pairing of greater than two chromosomes of each type (William and Barraclough, 2009). Increased ploidy level, particularly in conjunction with hybridization and resulting heterosis, enhance heterozygosity of an individual organism hence genetic variation within the population (reviewed in Horandl, 2009). All the above may lead to better tolerance of deleterious mutations due to higher number of homologous chromosomes and therefore the functional gene copies in asexuals. This view is further supported by a broader epigenetic landscape and narrow epigenetic resetting in polyploids and asexuals, for instance involving DNA methylation and small RNA pathways, and the enhanced epigenetic stability in clonal organisms might confer adaptive advantages (reviewed in Verhoeven and Preite, 2014). In addition, asexual reproduction provides short-term advantages for organisms primarily because the costs underlying the energy-consuming process of meiosis and involvement of two parents (twofold cost) are avoided (Dawson, 1995). Clonal reproduction may also be an advantage by fixing beneficial allelic combination in one organism. Extant ancient asexual species might confirm the evolutionary stability of this reproductive mode (Judson and Normark, 1996; Martens et al., 2003); however, the recent work uncovered that their evolution is based on horizontal gene transfer instead of meiotic sex (Eyres et al., 2015). It is equally important to note that most theories above utilized population or generation of microbes or fungus and/or simulations; the rate of mutation accumulation in clonal populations could as well be extremely slow in plastic organisms like plants (discussed later) across time scale and might provide advantage over sexuals in the wake of changing climatic conditions, as evident from the stable and wide-spread inter-continental occurrence of apomicts such as *Taraxacum* and *Hieracium*. Regulation of oxidative stress machinery was proposed as a switch between meiotic program as DNA repair and ameiotic reproduction in organisms with mixed (facultative) reproductive mode (Horandl and Hadacek, 2013). Nonetheless, selection of sexual versus asexual reproduction in organisms has remained an enigma and a stimulating research topic.

Gametophytic apomixis refers to asexual reproduction in plants, during which the sexual development is modified via avoidance of meiosis and fertilization (Winkler, 1908; Koltunow, 1993). Apomixis has been noted in about 0.1% of the flowering plants, leading to clonal reproduction through seeds *sensu stricto* (Mogie, 1992). Apomixis comprises four developmental processes: (a) alteration of meiosis known as apomeiosis, during which a diploid somatic cell termed as the Megaspore Mother Cell (MMC) undergoes only one cell division and ultimately yields an unreduced diploid egg cell (diplospory); or (b) avoidance of meiosis by a diploid somatic cell in the vicinity of MMC so that the former surpasses meiosis and mitotically develops a diploid egg cell (apospory); (c) formation of embryo from unfertilized egg cell, i.e., by parthenogenesis; and (d) autonomous (in the absence of fertilization) or sexual development of endosperm, which nourishes the parthenogenetic embryo (Koltunow, 1993; Hojsgaard et al., 2014b). These four developmental steps seem to be regulated independently, and genetic studies support both dominant and/or additive genetic effects underlying these traits. Apomixis generally occurs outside of the major cultivated plants, for instance, aposporous *Hieracium* and diplosporous *Taraxacum*; and several model systems have been established both in dicots and monocots, such that apomictic traits could possibly be transferred into the closest sexual relatives (Koltunow, 1993). Deciphering apomixis mechanisms in plants is a high priority research not only for evolutionary and developmental biologists, but also it is a long quest for plant breeders as it would allow propagation of elite clonal genotypes like F_1_ hybrids with superior breeding traits such as grain yield, biomass or timber, if apomictic traits are incorporated into the traditional breeding programs.

Following an initial observation in a wild apomict that led to a proposal that apomixis can be a mere deregulation of sexual developmental program, one of the widely accepted hypothesis is that the expression of genes controlling sexual reproduction may occur at the wrong place and/or the wrong time in apomicts (Nogler, 1984; Koltunow and Grossniklaus, 2003). In support of this hypothesis, transcriptome-wide changes of gene expression during apomixis events in the ovules or specific cell-types have been documented in various apomictic species (Hand and Koltunow, 2014). Expression of apomixis-specific gene fragments, for instance like those of *BABY BOOM* of apomictic *Pennisetum* (Conner et al., 2015) inducing partial parthenogenesis in sexual plants, or triple *Arabidopsis* meiotic mutants mimicking apomeiosis (d’Erfurth et al., 2009) lend support to a mutational model in which apomixis can be seen as mutation(s) modifying the sexual pathway.

In evolutionary terms, apomixis was proposed to have arisen due to hybridization between species that are inter-related, given that most apomicts are polyploids and maintain high degree of heterozygosity as means of buffering deleterious mutations (Ernst, 1918). Both hybridity and polyploidy presumably might have led to genome-wide effects, and might induce apomixis (Carman, 1997; Madlung et al., 2002; Bicknell and Koltunow, 2004; Wang et al., 2004). Although it is extremely rare, apomixis could also be sustained in diploid conditions, particularly when the apomictic populations represent diploid-aneuploids, or hybrids between two unrelated ecotypes or related species with differences in reproductive characters (Carman, 1997; Lovell et al., 2013). Apomictic polyploids were likely derived from initially unstable diploid apomictic hybrids, and they seem to feature high heterozygosity necessary for sustenance of apomixis due to dosage-dependent gene regulation leading to efficient management of deleterious mutations (Archetti, 2004). In addition, ploidy shifts lead to changes in epigenetic landscape, for instance in DNA methylation within several loci, and concomitant transcriptional reprogramming wired by the RNA-dependent DNA methylation (RdDM) pathways required for initiation and maintenance of apomictic traits (Verhoeven et al., 2010; Hand and Koltunow, 2014; Podio et al., 2014; Zappacosta et al., 2014). Thus, hybridization-derived polyploidy could sustain apomixis in plant populations. This view had lent support for the hybridization theory for evolution of apomicts (Carman, 1997), which suggests that apomixis might occur when two unrelated ecotypes or related species with differences in reproductive characters hybridized. The two distinct sets of genes from these hybrids would be asynchronously expressed, which might lead to precocious embryo sac initiation and parthenogenesis. Therefore, this theory is based on additive expression of sets of genes involved in reproduction, rather than mutations in genes involved in sexual reproduction, as evident in some apomictic species (Carman, 1997; Hojsgaard et al., 2014a). In summary, genetic control underlying apomixis in diploids versus polyploids remains largely unresolved, but it is conceivable that the molecular mechansims of evolutionary history traits related to hybridization, and/or ploidy and the environment (discussed below) would be indispensable for apomixis.

Environment is one of the important aspects that control organismal reproduction and fitness trade-offs. Due to their sessile nature, plants frequently encounter unfavorable abiotic conditions such as severe drought, extreme temperatures, contamination of soil by heavy metals and/or high salt concentration, and the same is true for a plethora of biotic conditions. Variation in parameters like temperature, light, water and nutrient levels act as stress agents and influence reproduction in plants (Hedhly et al., 2009; Thakur et al., 2010). Sexual reproduction is sensitive to stressful environmental conditions, and stress tolerance starting as early as in the vegetative phase and continuing into the reproductive phase is a key factor in sustainable plant productivity (Herrero and Johnson, 1980; De Storme and Geelen, 2014). The reproductive phase also gives the plant a chance to acclimatize to environmental variations. Genetic and epigenetic stress mechanisms have been extensively studied mainly in sexual plants more so during the sporophytic phase; data suggest that both vegetative and reproductive stress responses are inter-linked. For instance, under certain salinity thresholds, the female germline tissues of the sexual plant *Arabidopsis thaliana* seem to buffer stress much better than the male tissues, possibly due to a sink mechanism from the female tissues that are tightly dependent on the plant sporophyte (Sun et al., 2004). This proposition is in line with the role of maternal sporophytic tissue that could lend support for epigenetic, metabolic and genetic reprogramming in order to sustain apomictic events upon stress. It is important to note that apomictic hybrids and polyploids generally arose in stressful environments. Polyploids are generally known for their greater efficiency to tolerate abiotic stress such as osmotic stress, drought, light stress and to scavenge ROS species than the corresponding diploid individuals (Chandra and Dubey, 2010; Coate et al., 2013; Hao et al., 2013; Syamaladevi et al., 2016), and admittedly might offer a genomic haven to sustain apomixis in challenging environmental conditions (Lynch, 1984). Taken together, response to environmental factors such as abiotic stress acclimation is an important life-history trait, and might influence sexual and possibly apomictic reproduction.

*Boechera*, a close relative of the sexual model plant *Arabidopsis*, is a unique model system to study apomixis; the genus comprises of both diploid sexual, diploid and polypoid apomictic species, giving an opportunity to conduct comparative analyses of apomixis versus sex in the wild (Böcher, 1947, 1951; Roy, 1995; Naumova et al., 2001; Al-Shehbaz et al., 2006). Most apomictic *Boechera* are reproductively isolated, showing primary distribution in the western USA and Canada (Windsor et al., 2006). *B. stricta* (Graham) Al-Shehbaz (commonly known as Drummond’s Rockcress; hereafter referred to as line *Sex-1* in this work; 2n = 14) seems to be the most widespread sexual *Boechera*; repeated hybridization of this species likely paved way to reproductively isolated hybrids that ultimately resulted in the birth of apomictic species. Ploidy and zygosity are important for apomixis in *Boechera*, similar to the situation in most apomictic plants (Hand and Koltunow, 2014). The majority of the apomictic *Boechera* are triploids, and aneuploids, tetraploids and very rare diploids have also been identified (Roy, 1995; Schranz et al., 2006). In contrast to the high degree of inbreeding and homozygosity of sexual diploids, *Boechera* apomicts are highly heterozygous for most loci (Roy, 1995; Rushworth et al., 2011). Most apomictic *Boechera* are diplosporous, but rare mixed occurrence of apospory and diplospory have also been noted; parthenogenesis accompanies diplospory or apospory, and endosperm development is predominantly sexual (Böcher, 1947, 1951; Roy, 1995; Carman, 1997; Naumova et al., 2001; Lovell et al., 2013). *Boechera* genomes seem to have over 80% nucleotide sequence similarity to the genome of *A. thaliana*, making comparative analyses amenable. Recent work on *Boechera* uncovered genome-wide alterations of the transcriptomes from the reproductive lineage organs such as ovules or specific cell types such as the MMC, in diploid or triploid apomicts (Sharbel et al., 2010; Schmidt et al., 2014). These data, together with the unpublished data from our lab, support an unprecedented wave of transcriptional changes in the apomictic organ or cell-types of *Boechera*, hinting alterations in several transcription factors, meiotic and mitotic cell cycle machinery, hormonal regulation, DNA or histone methylation pathway etc., but a defined pattern of regulation has not been discernible. One of the intriguing classes of transcriptional changes noted in these transcriptome datasets is the abiotic and biotic stress category. It is interesting to note that in its native range varying between high altitude to coastal regions and deserts, *Boechera* species are often challenged by recurrent stress such as drought, heat, light, freezing etc.; thus highly prone to natural selection on stress tolerance traits (Roy, 1995; Franzke et al., 2011). Hence, *Boechera* has become a model plant to study particularly abiotic stress tolerance mechanisms (Rushworth et al., 2011; Gallas and Waters, 2015).

It is important to note that taxonomic revision within the *Boechera* genus is one of the daunting exercises for systematic botanists, as several *Boechera* species are known to repeatedly inter-hybridize, leading to novel species that are either sexuals or mostly facultative apomicts (Rushworth et al., 2011; Alexander et al., 2013). Several apomictic *Boechera* (diploid or polyploid) had been classified under *B. holboellii* clade and/or several others previously belong to this clade, e.g., *B. divaricarpa* (used in this study) had been reclassified into “trashcan” hybrids involving sexuals like *B. stricta* and *B. sparsifolia* (Rushworth et al., 2011). Diploids and polyploids (e.g., triploids) can be tractable within these “*holboeleii* and hybrid” clades (Naumova et al., 2001; Aliyu et al., 2010; Voigt-Zielinski et al., 2012), and current studies in several labs focus on the genome evolution of the identified apomictic diploids and their sexual parents by genome sequencing. Although exhaustive comparative studies have not yet been conducted, Roy concluded that *B. holboellii* apomictic populations are likely polyphyletic with substantial allelic variation thus greater fixed heterozygosity than what were observed for *B. gunnisoniana*, a stand-alone monophyletic apomictic triploid (Roy, 1995). We and others have used the latter for characterization of apomixis events (current study; Roy, 1995; Taskin et al., 2004; Schmidt et al., 2014; Kirioukhova et al., in preparation). *B. gunnisoniana* is not genome-sequenced, and no diploid individuals could be traceable in the wild. Unfortunately, the influence of hybridity but polyploidy on apomeiotic expressivity cannot be separated in this case, therefore, omic-datasets like Schmidt et al. (2014) or this study will have to be carefully compared and interpreted. As apomixis is also independent in evolution, independent apomictic *Boechera* species or clades might exhibit independent molecular signatures.

Given the importance of *Boechera* genus in evolutionary point of view, with its intrinsic characteristics to give rise to a number of independent populations with apomictic mode of reproduction and its origin from a habitat with elevated environmental stress, the main questions, which we address in this work are: (1) If there are specific gene expression alterations at the seedling stage, i.e., way in advance of the apomeiotic germline transition as a preparatory mechanism for apomixis-specific gene expression program, which could likely be continued at the reproductive stage of development. (2) In particular, we hypothesize that apomict-specific transcriptional shifts already in the seedling perhaps in light of genome-wide changes underlie (a) regulation of components of meiotic machinery, since the chosen diplosporous *Boechera* accessions exhibit a shortcut of meiotic division, and (b) stress-related regulatory networks as a response to harsh environmental conditions and hybrid genomic collisions predicted to have occurred during their evolution.

Using *Sex-1* as a baseline, we compared the seedling transcriptome of the obligate triploid apomict *B. gunnisoniana* (Rollins) W. A. Weber (commonly known as Gunnison’s Rockcress; hereafter referred to as line *Apo-1*). Both these strains were originally collected from a severe drought-prone gold mine high-elevation habitat in Colorado, USA (Roy, 1995) (Supplementary Figure S1). For further validations, we used a diploid apomict *B. divaricarpa* (A. Nelson) A. Löve and D. Löve of the *holboellii* hybrid clade; hereafter referred to as line *Apo-2*, which is evolutionarily distinct from *Apo-1*, and derived from a less-disturbed high-elevation habitat in Montana, USA (Schranz et al., 2005) (Supplementary Figure S1). Our RNA-seq approach revealed that, similar to apomictic ovules, apomictic seedlings show an array of transcriptional changes in several genes including down-regulation of two important meiotic regulators ASYNAPTIC 1 (ASY1) and MULTIPOLAR SPINDLE 1 (MPS1), accompanied by activation of stress-responsive genes particularly belonging to the NAC-DOMAIN CONTAINING (NAC) transcription factor family and LATE EMBRYOGENESIS ABUNDANT (LEA) family. Furthermore, we show evidence that osmotic stress, as an environmental factor; and DNA methylation, as an epigenetic modifier, seems to be involved in gene regulation during apomictic seedling development. Together, we propose a model integrating stress response machinery as a developmental buffer from early seedling development and simultaneously co-evolved apomeiosis/apomixis-specific regulatory network under polyploidy. In line with hypothesis of stress-related background of sex (Hadany and Beker, 2003a,b; Horandl and Hadacek, 2013), our findings suggest that the shift in the stress-response regulation might have created stress tolerant genomic environment for successful apomeiotic transition in *Boechera*.

## Materials and Methods

### Plant Material and Ploidy Analysis

*Boechera* seeds (sexual and asexual) were generous donations of Bitty Roy (University of Oregon, USA) and Eric Schranz (Wageningen University, USA); seeds were originally isolated from the native habitat of *Boechera* in the USA (Supplementary Figure S1), and they represent green-house or growth-chamber grown bulked seeds when donated. The progeny were further screened by bulked and single seed flow cytometry to remove odd genotypes (4n, 6n, aneuploids), and 3n and 2n plants of *Apo-1* and *Apo-2* were identified and characterized by cytology (unpublished data). Subsequent generation of seeds were surface-sterilized, stratified at 4°C and grown on half-strength MS agar plates for 17–18 days. For reproductive development, they were transferred subsequently to soil (Profi-substrate, Einheitserde, Germany) and quartz sand in a 4:1 combination. Seedlings and plants were grown in dedicated plant growth chambers with long day photoperiod (16-h light/8-h darkness cycle), under cool white light with ca. 120 μmol m^-2^s^-1^ intensity, 60–65% air humidity and 22°C. Plants were ploidy genotyped either in a Partec flow cytometer (Sysmex, Germany) or in a Guava^®^ easyCyte flow cytometer (Millipore, USA).

### mRNA-seq Analyses

Seventeen to eighteen days old seedlings from *Sex-1* and *Apo-1* accessions were subjected to mRNA sequencing. Seedlings were snap-frozen and kept at -80°C until RNA extraction; tissue was ground using a tissue lyser (Retsch, Germany). RNA extraction was performed using QIAGEN RNeasy Mini Kit and DNase–treated according to manufacturer’s instructions (Qiagen, USA). The RNA quality was further validated in a Bioanalyzer (Life Technologies, USA). mRNA transcripts purification, cDNA synthesis, library preparation and NGS sequencing were performed as per the routine pipeline established at the facility of Fasteris, Geneva, Switzerland. Illumina HiSeq2000 was used to carry out paired-end sequencing generating a minimum of 100 bp per read.

### Gene Expression Analysis and GO Annotation

All reads from Illumina sequencing (ArrayExpress accession: E-MTAB-4972) were first quality-assessed using FastQC (Andrews, 2010) and mapped to *Arabidopsis* (TAIR-10) genome using Tophat-2.0.9 (Kim et al., 2013). The percentage of reads mapped from *Sex-1* library were 33.0% and from *Apo-1* library 34.9%, respectively. To quantify transcript abundances and differential expression analysis, Cuﬄinks-2.1.1 and Cuffdiff-2.1.1 tools were used with upper quartile normalization and multi-read correction was applied to procure fragments per kilobase of transcript per million mapped reads (FPKM) values (Trapnell et al., 2010, 2012). In parallel to obtaining a list of differentially expressed genes based on corrected *p*-values from cuffdiff (*N* = 87), FPKM values from cuﬄinks file were used to generate the second list of differentially expressed candidate genes based on twofold changes between expression values across both genotypes. It must be noted that mapping *Boechera* reads to *Arabidopsis* genome (80% similarity) is of heterologous nature, therefore, we relaxed the filtering criteria and categorized genes showing mis-regulation by twofold change. Based on this list, we found significant number of genes (∼4100) to be differentially expressed across both genotypes. The list of differentially expressed genes was then subjected to GO enrichment analysis using BiNGO tool from Cytoscape after Benjamini and Hochberg false discovery rate (FDR) correction with *p*-value cut-off of ≤0.05 (Maere et al., 2005). Gene ontology and annotations were improved with data acquired from the following websites: www.arabidopsis.org; http://planttfdb.cbi.pku.edu.cn; http://ahd.cbi.pku.edu.cn, where appropriate.

### Osmotic Stress (*In vitro* Assay), Drought Test and DNA Methylation Inhibitor Treatment

*In vitro* osmotic stress treatment was carried out using half-strength Murashige and Skoog (½MS) agar plates infused with PEG-8000 (polyethylene glycol). Once the MS media was solidified, PEG was overlaid and allowed to equilibrate overnight. Two concentrations of PEG (400 and 550 g/L) were tested on *Boechera* seedlings. Prior to transferring seedlings, PEG solution was poured according to (Verslues et al., 2006). Since 550 g/L PEG almost completely inhibited the root growth, 400 g/L PEG was used for final experiments. For salt (osmotic + ionic) stress, ½MS agar plates supplemented with 50 mM NaCl were used as this concentration causes a similar effect on primary root elongation in *Arabidopsis* (Verslues et al., 2006). Individual seedlings (*n* = 20 seedlings each for mock and PEG stress treatment in four replicates, and *n* = 8 for salt stress treatment) grown for 11–12 days on mock ½MS agar plates in vertical position were then transferred to both stress treatment and mock plates, arranged in a manner to have the same start point for further growth of roots and grown for next 7 days. Each plate contained seedlings from all three genotypes, and the experiments were replicated at least four times to avoid plate effect. Following the seventh day of treatment, images were captured and primary root length measurements were carried out using the software ImageJ (Schneider et al., 2012). Drought treatment of flowering plants pre-grown at control conditions was performed by stopping watering for 9 days until soil in pots was almost dry and some plants showed first signs of wilting, and then resuming watering to maintain the acquired weight of the pots and prevent plants drying out.

Zebularine, a chemical analog of the cytosine nucleoside that efficiently inhibit DNA methyltransferase activity (Baubec et al., 2009) was used as a DNA methylation inhibitor. Different concentrations of zebularine (50–175 μM) were first tested for their growth effect on *Boechera* seedlings *in vitro*. For final experiments, we used 175 μM zebularine treatment, as the seedlings still retained chlorophyll but their growth was inhibited. Seedlings (*n* = 10 each for control and for treated) grown on ½MS agar plates for 11–12 days were transferred to ½MS mock agar plates or supplemented with 175 μM zebularine. Arrangement of seedlings in the plates was similar to that of PEG treatment. Images were recorded and samples were snap-frozen for RNA allowing 7 days of growth upon exposure to the nucleoside.

### RNA Extraction, cDNA Synthesis and qPCR Validation

Seedlings grown on vertical ½MS agar plates, and premeiotic buds from plants grown on soil in control conditions were collected and immediately snap-frozen for RNA extraction. Developmental staging of pre-meiotic flower buds used for gene expression validation was determined based on clearing of ovule samples by DIC microscopy. The bud size and images of corresponding ovule development for each genotype are furnished in Supplementary Figure S3. RNA isolation and cDNA synthesis were performed as described previously (Johnston et al., 2008, 2010). In brief, frozen tissues were ground similarly to the RNA-seq experiment; RNA was extracted using Trizol method (Chomczynski, 1993) and treated with DNase I; reverse transcription was done with SuperScript III First-Strand Synthesis System, according to the manufacturer instructions (Thermofisher, USA). To validate RNA-seq results, qRT-PCR of selected genes with strongly increased or decreased expression in the apomict was carried out. Three biological replicates of three seedlings each in three technical replicates were used for all control seedlings; similar experimental setup was also used for qRT experiments on premeiotic buds and zebularine-treated seedling experiments. The list of candidates included genes from meiotic and stress machinery. Primer sequences are listed in Supplementary Table S5. SYBR Green assays were performed with StepOnePlus Real-Time-PCR System (Applied Biosystems, USA) and normalized as described in (Pellino et al., 2011). Calculations of relative expression levels and generation of graphs was carried out using Microsoft Excel 2010.

### Morphological Analysis of Reproductive Development

Pre- and post-meiotic ovules were fixed in ice-cold 9:1 ethanol:acetic acid fixative and incubated overnight at 4°C, then re-hydrated in 90, 80, and 70% ethanol series. Ethanol was replaced with clearing solution (8:2:1 chloralhydrate:glycerol:water) for 12–16 h at 4°C, and ovules were dissected out on microscopy slides with help of insulin needles in a drop of clearing solution, and mounted under cover slips. Differential interference contrast (DIC) imaging was performed on a Leica DMI6000 microscope.

### Venn Diagrams and Image Processing

Venn diagrams were made with online Venn diagram generator available at http://bioinformatics.psb.ugent.be/webtools/Venn. Images were treated in Adobe suite of software such as Illustrator or Photoshop CS for quality of representation only.

### Statistical Analysis

Quantitative data were analyzed using one-tailed unpaired Student’s *t*-test for comparisons between groups. In addition, significance of differential gene expression response to treatments was evaluated using two-way ANOVA. Significance levels used were α ≤ 0.01 (^∗∗^) and α ≤ 0.05 (^∗^). Calculations were carried out using Microsoft Excel 2010 and http://vassarstats.net/anova2u.html.

## Results

### Meiosis, Stress and Hormonal Regulation Are Commonly Affected Signaling Hubs in Apomictic Seedling Transcriptome

**FIGURE 1:**
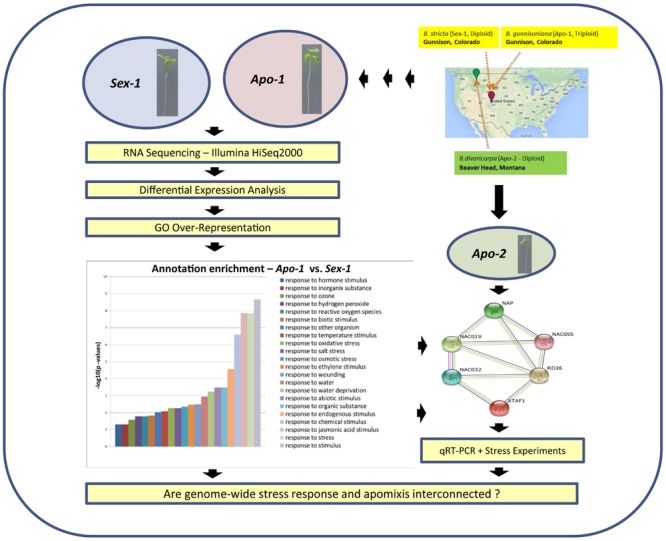
**A flow chart describing the transcriptome approach and validation in order to identify stress-specific changes underlying apomixis at seedling stage.** Seedlings of *Sex-1* and *Apo-1 Boechera* strains were subjected to mRNA sequencing followed by differential gene expression analysis and identification of GO (Gene Ontology) enrichment for stress. The bar chart represents –log_10_ values of corrected *p*-values for stress-related annotations obtained after GO enrichment using Bingo tool from Cytoscape (http://www.cytoscape.org). *Apo-2* accession was used as a secondary apomictic genotype for real-time qRT-PCR-based validations and stress experiments.

We conducted a transcriptome-wide differential gene expression analyses between a sexual diploid, and an apomictic triploid *Boechera* that originated presumably through hybridization and polyploidization in the same stress-prone habitat (**Figure 1**, Supplementary Figure S1). We performed comparative RNA-seq analyses between the apomictic seedlings versus the sexual seedlings, in order to uncover the molecular signatures associated with the evolutionary history that might have formed the basis of evolution and maintenance of apomixis. A little over 3000 *Boechera* genes were found differentially expressed between *Apo-1* and *Sex-1* seedlings, accounting for about 15% of the total number of corresponding *Arabidopsis* loci qualified for differential analyses upon heterologous mapping. From the analysis of gene ontology (GO) enrichment of these genes we noted that response to stimulus and stress categories predominated, ranging up to 23% of the total (**Figure 1**, Supplementary Table S1). Both biotic and abiotic GO stress categories were present; and abiotic stress categories were frequented by response to osmotic, drought and salt stress categories. Other primary categories included response to wounding, hormones, ethylene, oxidative stress, metabolic processes etc. Regardless of lists of genes upregulated or down-regulated in the apomictic seedlings, the overall annotation enrichment helped us to pinpoint the essence of stress being an important category to distinguish the vegetative transcriptome of the apomict over the corresponding sexual (see the bar chart in **Figure 1**; Supplementary Figures S1 and S2). Intriguingly, a total of 12 genes associated with meiosis were found differentially expressed in apomictic seedlings (**Table 1**). We validated our transcriptome data by a two-fold approach: (a) comparison of real-time qPCR-determined expression levels of representative candidate genes not only between *Apo-1* and *Sex-1*, but also in another apomict, *Apo-2*, which is distinct from *Apo-1* in terms of ploidy and evolutionary history, and (b) testing deregulation of meiotic and stress response genes also in reproductive organs at onset of (apo)meiosis.

**Table 1:** Homologs of meiotic genes deregulated in apomictic seedlings of *Boechera.*

*Arabidopsis* locus ID	Locus name	*Sex-1* FPKM	*Apo-1* FPKM	Log_2_ Fold Change
**Down-regulated in *Apo-1***				
*AT1G67370*	*ASYNAPTIC 1 (ASY1)*	6.84	0.51	-3.74
*AT3G20475*	*MUTS-HOMOLOGUE 5 (MSH5)*	2.89	0.51	-2.50
*AT3G52115*	*GAMMA RESPONSE GENE 1 (GR1)*	1.20	0.09	-3.80
*AT4G14180*	*PUTATIVE RECOMBINATION INITIATION DEFECT 1 (PRD1)*	1.85	0.72	-1.36
*AT4G35520*	*MUTL PROTEIN HOMOLOG 3 (MLH3)*	2.48	1.14	-1.12
*AT5G57880*	*MULTIPOLAR SPINDLE 1 (MPS1)*	11.57	3.01	-1.94
**Up-regulated in *Apo-1***				
*AT4G29170*	*MEIOTIC NUCLEAR DIVISION PROTEIN1 (MND1)*	1.01	4.35	2.11
*AT4G00020*	*BREAST CANCER 2 LIKE 2A (BRCA2(IV))*	0.30	0.85	1.51
*AT5G01630*	*BRCA2-LIKE B (BRCA2B)*	0.41	1.14	1.46
*AT5G57450*	*HOMOLOG OF X-RAY REPAIR CROSS COMPLEMENTING 3 (XRCC3) (XRCC3)*	0.51	1.23	1.28
*AT3G27730*	*ROCK-N-ROLLERS (RCK)*	0.19	0.45	1.25
*AT4G17380*	*MUTS-LIKE PROTEIN 4 (MSH4)*	1.55	3.35	1.11

**FIGURE 2:**
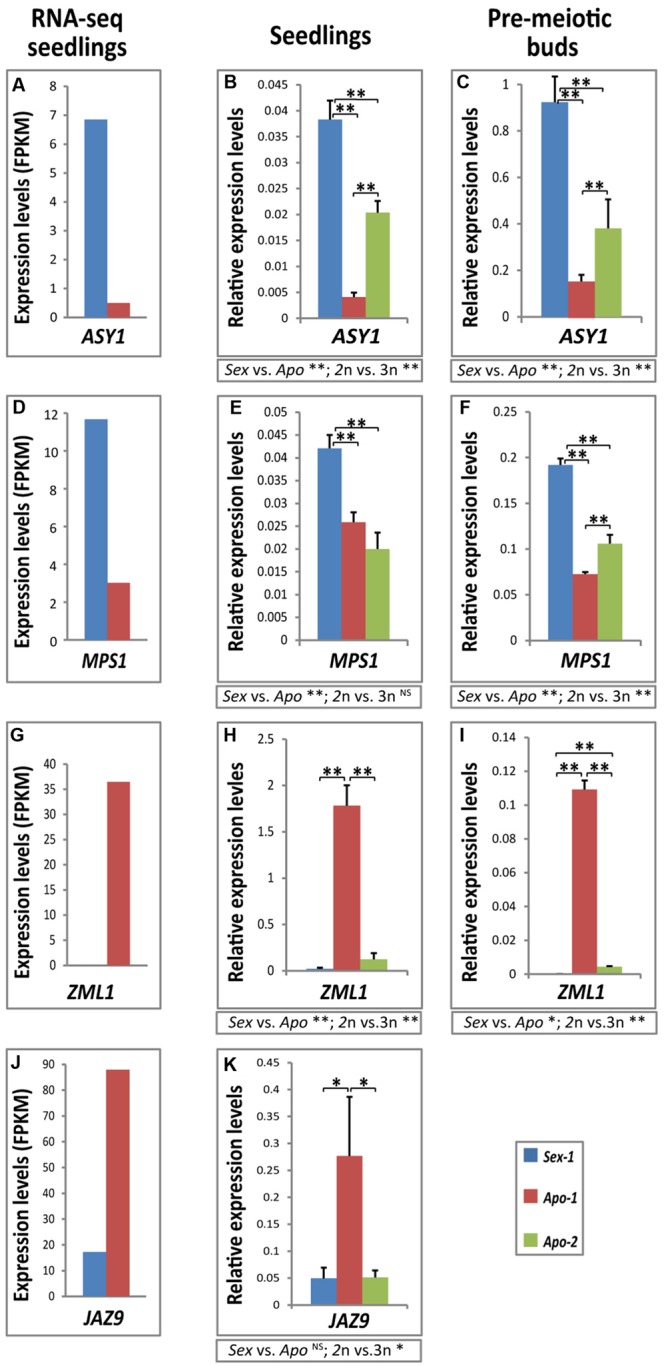
**Differential real-time quantification of gene expression validates our transcriptome approach.** Demonstrated here is down-regulation of homologs of meiotic genes *ASY1* **(A–C)** and *MPS1* **(D–F)**, accompanied by upregulation of stress-response genes, a GATA type (ZML1) transcription factor **(G–I)** and *JASMONATE-ZIM-DOMAIN PROTEIN 9* (*JAZ9*) **(J,K)**. *Sex-1* versus *Apo-1* seedlings-derived RNA-seq differential expression data (first column) were validated by real-time qRT-PCR (second column), and the expression analyses were further assayed by qRT-PCR in corresponding floral buds of the pre-meiotic MMC-stage (Supplementary Figure S3) (third column). *Apo-2* was used as an additional apomictic genotype for qRT-PCR comparisons. FPKM – fragments per kilobase per million. Statistical significance was calculated using t-test; significance levels: ^∗∗^α ≤ 0.01; ^∗^α ≤ 0.05; ^NS^, not significant. *t*-test results for comparisons between groups for reproductive mode and ploidy (*Sex* vs. *Apo*, and diploid vs. triploid) are indicated below the respective bar chart.

Consistent with our RNA-seq data (**Table 1**, Supplementary Table S3, **Figures 2A,D**), we could confirm that homologs of *ASYNAPTIC 1* (*ASY1*) and *MULTI-POLAR SPINDLE 1* (*MPS1*) were significantly down-regulated in seedlings of *Apo-1* compared to *Sex-1* (**Figures 2B,E**) as well as at pre-meiotic stage in flower buds (**Figures 2C,F**, Supplementary Figure S3). Between *Apo-1* and *Apo-2* seedlings, *ASY1* level was lower in *Apo-1* than *Apo-2* while *MPS1* showed insignificant differences between the two apomicts. Similar trend was also observed in premeiotic flower buds. In order to subtract ploidy from reproduction mode-related effects, we compared the gene expression in groups of the diploids with triploid (2n vs. 3n) and the sexual with apomicts (*Sex* vs. *Apo*). When mean seedling expression value of both apomicts was compared to that of the sexual, *MPS1* expression was found significantly decreased, and *ASY1* displayed a similar downregulation trend. However, ploidy groups also exhibited significant difference in gene expression, and *MPS1* expression in seedlings was tested as ploidy-independent (**Figure 2E**). Meiotic gene deregulation was further accompanied by upregulation of gene homolog of an abiotic (light) stress response GATA TRANSCRIPTION FACTOR 24 (GATA24) (Supplementary Table S2), otherwise annotated as ZIM-LIKE 1 (ZML1) in *Arabidopsis*, both in apomictic seedling tissues and flower buds of *Apo-1* and *Apo-2* (**Figures 2G–I**). However, *ZML1* levels were much higher in *Apo-1* compared to *Apo-2* in both the stages and probably reflected ploidy-specific gene expression pattern. Similarly, a biotic stress-specific gene *JASMONATE-ZIM-DOMAIN PROTEIN 9* (*JAZ9*) was found activated in *Apo-1* only (**Figures 2J,K**, Supplementary Table S2). *ZML1* showed both significant effect between reproductive and ploidy groups, but *JAZ9* expression seemed to be rather ploidy-dependent. Together, a subset of meiotic and stress-specific genes was deregulated in the apomictic seedlings and premeiotic buds, thus confirming our RNA-seq data and a possible association with the reproductive stage. This finding let us to propose that the identified transcriptional modifications in an early stage could likely follow throughout apomictic development. Accession-specific differences between the two apomicts could also be noticed pointing at the role of ploidy and/or evolutionary history in gene expression, yet suggesting their role preferentially in the apomicts. The above validations support that our dataset of about 700 stress- and stimulus-associated genes differentially expressed in the asexual over sexual seedlings could be resourceful for connecting stress, ploidy and reproductive nodes of gene regulatory networks in development.

**FIGURE 3:**
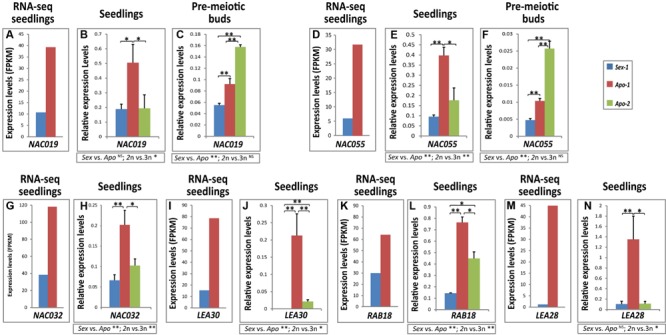
**Transcripts of stress-related NAC transcription factors and LEA-type storage protein homologs are upregulated in the apomict.** Histograms showing real-time qRT-PCR results for **(A–C)** *NAC019*, **(D–F)** *NAC055*, **(G,H)** *NAC032*, **(I,J)** *LEA30*, **(K,L)** *RESPONSIVE TO ABA 18 (RAB18)* and **(M,N)** *LEA28.* The initial RNA-seq data used in validation are graphically represented in **(A,D,G,I,K,M)**. Statistical significance calculated by *t*-tests is indicated by asterisks. *t*-test results for comparisons between groups for reproductive mode and ploidy (*Sex* vs. *Apo*, and diploid vs. triploid) are indicated below the respective bar chart. Statistical significance levels: ^∗∗^α ≤ 0.01; ^∗^α ≤ 0.05; ^NS^not significant.

A careful examination of our dataset indicated that transcription factor deregulation is indeed one of the major components of the apomict/stress-specific transcriptome accounting for about 15% (Supplementary Tables S1 and S2). Whereas members of the AP2/EREBP (APETALA2/ethylene-responsive element binding proteins) transcription factor family predominated equally in both upregulated and down-regulated genes in apomictic seedlings, transcription factors belonging to the NAC [NAC domain: NAM (for NO APICAL MERISTEM), ATAF 1 and 2, and CUC2 (for CUP-SHAPED COTYLEDON 2)], bZIP (BASIC LEUCINE ZIPPER) and MYB (R2R3-type) families were enriched in the upregulated gene lists (**Table 2**, Supplementary Tables S2 and S4). bHLH (BASIC HELIX-LOOP-HELIX), WRKY (WRKY DNA-BINDING PROTEIN) and C2H2-type Zn-Finger transcription factor families were exclusively abundant in the down-regulated category (Supplementary Table S2). Since the stimulus, hormonal control and transcription factor regulation categories are tightly inter-connected; we examined if stress-related hormonal regulatory features could be identified as specific for *Apo-1* seedlings transcriptome. We noticed that nearly one-third of differentially expressed genes (26 and 36% of down-regulated and upregulated, respectively) respond to hormones such as abscisic acid (ABA), auxin, jasmonic acid (JA), ethylene, salicylic acid (SA), gibberellic acid (GA) and cytokinin (Supplementary Table S2). It is interesting to note that genes of the auxin biosynthesis and response [e.g., *YUCCA8, CYTOCHROME P450 CYP79B2, INDOLE-3-ACETIC ACID INDUCIBLE 5* (*IAA5*), *IAA19, IAA32*], cytokinin response [e.g., *CYTOKININ INDEPENDENT 2* (*CKI2*), *TYPE-A RESPONSE REGULATOR* (*ARR4*), *ARR5, Arabidopsis HISTIDINE-CONTAINING PHOSPHOTRANSFER FACTOR 5* (*AHP5*)] regulatory members are generally down-regulated, in contrast to those that belong to biosynthesis and response of ABA [e.g., *NINE-CIS-EPOXYCAROTENOID DIOXYGENASE 3* (*NCED3*); *RESPONSIVE TO ABA 18* (*RAB18;* **Figures 2C,D**); *ABSCISIC ACID RESPONSIVE ELEMENTS-BINDING FACTOR 2* (*ABF2*), *DRE-BINDING PROTEIN 2A* (*DREB2A*), *RD26 NAC*] and the JA pathway [*LIPOXYGENASE 2* (*LOX2*), *FATTY ACID DESATURASE 8* (*FAD8*), *ALLENE OXIDE SYNTHASE* (*AOS*), *JAZ9*] were upregulated in the apomictic *Boechera* seedlings (**Table 2**, Supplementary Table S2). The antagonistic situation between different hormone responses, enrichment of specific hormone regulation categories of ABA and JA, and upregulation of several genes coding for the LATE EMBRYOGENESIS ABUNDANT (LEA)-type storage proteins [e.g., *LEA30, RAB18* and *LEA28* (**Figures 3J–N**)] suggest that the apomictic seedlings likely have better stress acclimation than the sexual seedlings, perhaps in a ploidy-dependent manner.

**Table 2:** NAC-DOMAIN CONTAINING (NAC) transcription factor homologs deregulated in apomictic seedlings of *Boechera.*

*Arabidopsis locus ID*	Locus name	*Sex-1* FPKM	*Apo-1* FPKM	Log_2_ Fold Change
**Up-regulated in *Apo-1***				
*AT4G28530*	*NAC074*	0.11	5.53	5.63
*AT1G69490*	*NAC-LIKE, ACTIVATED BY AP3/PI (NAP)*	3.41	29.26	3.10
*AT3G15500*	*NAC055*	4.45	32.40	2.87
*AT1G76420*	*CUP SHAPED COTYLEDON3 (CUC3)*	0.14	0.92	2.69
*AT2G46770*	*NAC043*	0.11	0.59	2.43
*AT4G27410*	*RESPONSIVE TO DESICCATION 26 (RD26)*	21.49	115.67	2.43
*AT5G17260*	*NAC086*	0.21	1.02	2.28
*AT3G44290*	*NAC060*	1.98	9.26	2.22
*AT5G53950*	*CUC2*	0.13	0.58	2.11
*AT5G64060*	*NAC103*	0.45	1.64	1.88
*AT1G52890*	*NAC019*	10.52	38.15	1.86
*AT1G01720*	*NAC002*	29.54	103.70	1.81
*AT5G66300*	*NAC105*	0.79	2.55	1.69
*AT1G77450*	*NAC032*	38.82	117.85	1.60
*AT2G27300*	*NTM1-LIKE 8 (NTL8)*	2.36	6.27	1.41
*AT5G07680*	*NAC080*	1.22	3.24	1.41
*AT3G18400*	*NAC058*	2.04	5.12	1.33
*AT5G56620*	*NAC099*	0.53	1.30	1.28
*AT2G24430*	*NAC038*	5.71	13.80	1.27
*AT5G61430*	*NAC100*	2.97	7.09	1.26
*AT1G32510*	*NAC011*	0.19	0.41	1.11
*AT1G02250*	*NAC005*	1.01	2.01	1.00
**Down-regulated in *Apo-1***				
*AT1G01010*	*NAC001*	3.95	0.76	-2.38
*AT1G26870*	*NAC009*	0.84	0.13	-2.70
*AT1G32770*	*NAC012*	0.87	0.31	-1.48
*AT1G33280*	*NAC015*	2.09	0.94	-1.16
*AT1G52880*	*NO APICAL MERISTEM (NAM)*	9.81	4.54	-1.11
*AT2G17040*	*NAC036*	45.16	5.77	-2.97
*AT2G33480*	*NAC041*	7.24	3.09	-1.23
*AT2G43000*	*NAC042*	8.11	0.60	-3.77
*AT3G12977*	*NAC-like*	12.56	5.32	-1.24
*AT3G15510*	*NAC056*	15.65	6.03	-1.38

### Stress-Responsive NAC Transcription Factors are Modulated Prior Apomeiotic Transition

Among the several stress-responsive transcription factors identified to be deregulated in the apomict (discussed above), we chose to examine the NAC transcription factors further. **Table 2** enlists all the NAC factors uncovered by our RNA-seq approach. Particularly six *Boechera* protein homologs of the *Arabidopsis* NACs, NAC019, NAC032, NAC055, RD026, NAP and ATAF1 (**Table 2**, Supplementary Table S4) that presumably might function as well during apomeiotic transition, could be mapped within a probable regulatory network in *Arabidopsis*, as pinpointed by the String database (**Figure 1**) ^1^. We examined if some of these *NAC* homologs were indeed differentially expressed. *Boechera* homologs of *NAC019* and *NAC055* showed significant upregulation both in the apomictic seedlings and in the apomeiotic flower buds of *Apo-1* (**Figures 3A–F**). *NAC032* confirmed the trend from transcriptome data and showed upregulation in *Apo-1* seedlings as well. Although these genes did not show significant upregulation in the seedlings of *Apo-2*, the corresponding apomeiotic floral buds of *Apo-2* showed stronger enrichment than *Sex-1* and *Apo-1* (**Figures 3C,F**) exhibiting a significant expression increase of the average between apomicts. The expression changes for *NAC019, NAC055* and *NAC032* between *Apo-1* and *Apo-2* were also found to be significantly different. Although there were certain differences in *NAC* expression profiles between the apomicts at early developmental stages, which could concern rather difference in ploidy level or genetic background, *NAC055* and *NAC019* expression in flower buds was not different between ploidy groups but only significantly reproductive-mode-dependent. Together, *NAC* gene family upregulation may represent a key unifying background for early stress response, in apomictic *Boechera* species.

### Osmotic Stress Experiment Reveals that Apomictic Seedlings Efficiently Buffer Stress Conditions

**FIGURE 4:**
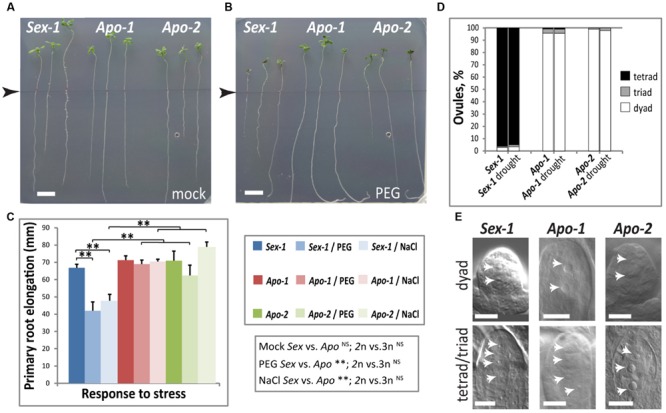
**Osmotic stress effects on early seedling and reproductive stages of development in *Boechera*. (A–C)** An *in vitro* primary root elongation assay demonstrates that the apomicts buffer osmotic stress better than the corresponding sexual. Representative photographs of *Boechera* seedlings grown without **(A)** and with osmotic stress imposed by an osmoticum, polyethylene glycol (PEG8000, 400 g/L) **(B)**. Root length measurement start points are marked by arrow-heads in both images. **(C)** Quantification of root elongation in the presence of PEG, and an additional osmoticum NaCl (50 mM). Statistical significance verified by *t*-test is indicated by asterisks. *t*-test results for comparisons between groups for reproductive mode and ploidy (*Sex* vs. *Apo*, and diploid vs. triploid) are indicated below the respective bar chart. **(D)** Quantification of meiotic versus apomeiotic events (tetrads and dyads, respectively) in post-meiotic/apo-meiotic ovules of plants that were well-watered (*N* = 107, 117, 95, respectively) versus those that underwent moderate drought stress (*N* = 59, 91, 68, respectively), did not reveal obvious differences **(E)**. Representative differential interference contrast (DIC) images of cleared ovules quantified in **(D)**. Scale bars in **(A,B)** 10 mm, in **(E)** 10 μm. Statistical significance levels: ^∗∗^α ≤ 0.01; ^NS^not significant.

To understand stress sensitivity of *Boechera* species, we carried out *in vitro* osmotic stress treatments in line with the output from transcriptome analysis of *Boechera* seedlings featuring stress signatures such as drought, salinity and osmotic stress tolerance. We treated *Boechera* seedlings with PEG-8000 (polyethylene glycol 8000), using plate assays, generating changes in osmotic balance and therefore decreasing water availability as a major component of drought stress (Verslues et al., 2006). We tested multiple concentrations of PEG for *Boechera* growth and found that PEG 400 g/L would impose moderate osmotic stress; PEG 550 g/L seemed to be too harsh for the seedlings as most of the exposed plants did not survive well, regardless of accessions (data not shown). Alteration in osmotic conditions had a noticeable root growth effect in *Sex-1* roots but not in the apomicts. Primary root elongation of the *Sex-1* seedlings treated with PEG 400g/L was found to be smaller by ∼25% in comparison to mock treated *Sex-1* seedlings (**Figures 4A–C**). However, for both *Apo-1* and *Apo-2* seedlings, no significant difference was observed in primary root lengths in either of the treatments, suggesting the prevalence of a better osmotic stress tolerance mechanism in both apomicts than in the sexual. This finding was further corroborated by the differential response of the sexual and apomicts to salt concentrations that cause both osmotic and ionic stress using 50 mM NaCl which caused a very similar effect decreasing the primary root growth exclusively in the sexual (**Figure 4C**). Whereas primary root extension was similar in the control treatment across all *Boechera* accessions, only the sexual exhibited stress-induced effect, and both osmotic and salt stress caused significantly different effect between reproductive groups but that was not ploidy-dependent. In addition to the *in vitro* assays, we enforced moderate drought on soil grown plants in standard growth room conditions at 22°C to understand if osmotic stress induced by drought impact the overall growth and reproduction of the three genotypes. We analyzed the ratio of reproductive modes in ovules upon drought stress, asking if stress impacted meiosis versus apomeiosis. The proportion of rare sexual-like triads and the routine meiotic tetrads in relation to apomeiotic dyads did not detectably change upon drought exposure in both apomicts (**Figure 4**). In sum, apomictic seedlings seem to be better acclimated to osmotic and ionic stress in seedlings, but stress does not significantly shift obligate apomeiotic mode to sexual mode during the reproductive phase.

### Does DNA Methylation Affect Gene Regulation in Apomictic Seedlings?

**FIGURE 5:**
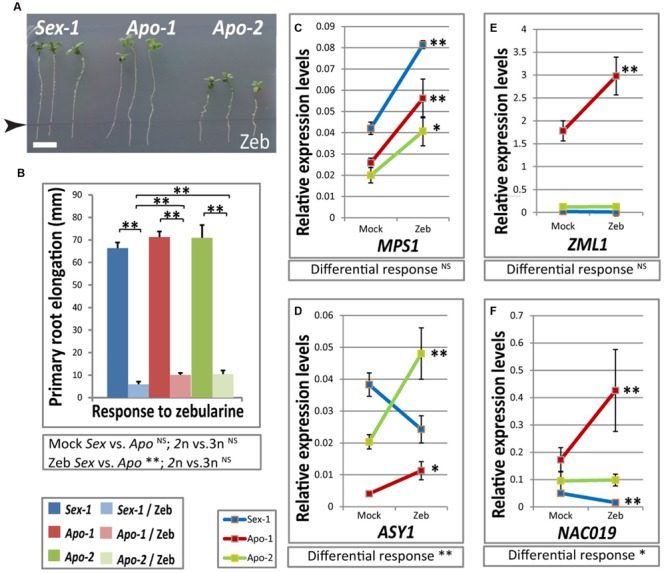
**DNA de-methylation agent, zebularine, has impact on primary root growth, regulation of meiotic genes and abiotic stress factors in *Boechera*. (A)** A representative photograph of *Boechera* seedlings upon *in vitro* application of a DNA methylation inhibitor, zebularine, exhibiting strong root growth retardation (for a comparable representative control, see **Figure 4A**). Note that zebularine (175 μM) inhibited growth in all three genotypes. **(B)** Zebularine-treated sexual plants had significantly shorter primary root elongation when compared to that of both apomicts. Statistical significance verified by *t*-test is indicated by asterisks. *t*-test results for comparisons between groups for reproductive mode and ploidy (*Sex* vs. *Apo*, and diploid vs. triploid) are indicated below the respective bar chart. **(C–F)** Real-time qRT-PCR-based gene expression of *MPS1* **(C)**, *ASY1* **(D)**, *ZML1* **(E)** and *NAC019* **(F)** homologs in three *Boechera* genotypes upon zebularine treatment. Significance of differential statistical response between genotypes verified by two-way ANOVA is given below each graph. Scale bar in **(A)** 10 mm. Statistical significance levels: ^∗∗^α ≤ 0.01; ^∗^α ≤ 0.05; ^NS^not significant.

Our transcriptome approach uncovered the parallel alterations between stress and meiosis regulation in the apomictic *Boechera*. Unpublished data from our lab and meta-analysis of our transcriptome data to large scale databases indicated definite role of methylation machinery in apomixis specific gene expression changes. We indeed observed that the *Arabidopsis* homologs of several of our stress-specific candidate transcription factors and meiotic genes are likely epigenetically regulated in seedlings via DNA methylation and/or small RNAs, or repressive histone methylation marks (Supplementary Tables S2–S4). In the absence of full genome and methylome sequences of all three species studied here, we conducted a DNA methylation inhibitor assay using a demethylation agent zebularine (Baubec et al., 2009), to identify gene expression changes associated with global DNA methylation. Zebularine exposure resulted in significant effects on all genotypes causing strong decrease in root length (**Figure 5A**, compare to **Figure 4A**), due to the genome-wide demethylation effect of this nucleoside combined with its redox-active nature, similar to growth retardation of *Arabidopsis* when applied *in vitro.* It must be noted, however, that there was small but significant difference in response upon zebularine treatment; both apomicts retained a slightly better root elongation than the sexual (**Figure 5B**). We examined the effect of this DNA methylation inhibitor on selected candidate genes by real-time qRT-PCR comparing zebularine-treated versus mock-treated seedling gene expression of meiotic genes: *ASY1* and *MPS1*; and a subset of stress-responsive transcription factors: *ZML1, NAC019, NAC032* and *NAC055*. The first four candidates seem to be regulated by DNA methylation as their expression was affected by zebularine exposure (**Figures 5C–E**), and both *NAC032* and *NAC055* did not show similar regulation (data not shown). The qRT-PCR results showed some interesting changes in expression of the candidate genes. *MPS1* seems to be repressed by DNA methylation in all species regardless of the reproductive mode, as there was no significant difference in response to zebularine application between the *Boechera* accessions used (**Figure 5B**). *ASY1, ZML1* and *NAC019* expression upon zebularine treatment was either unaltered or down-regulated in the sexual seedlings but they were strongly activated in *Apo-1*, and *ASY1* upregulation seem to be common in both apomicts and different from the sexual (**Figures 5C–F**). *ASY1* and *NAC019* expression responses to zebularine were significantly different between species based on two-way ANOVA test, while *ZML1* exhibited rather ploidy-dependent effect. Our data suggest locus-specific gene expression changes upon induced global DNA demethylation coupled with cellular stress distinguish between both ploidy groups and apomictic and sexual genotypes.

## Discussion

### *Boechera*, an Ideal Model for Studying Co-regulation of Apomixis, Polyploidy and Environmental Response

*Boechera* is one of the primary plant model systems to study evolution and regulation of apomixis associated traits such as meiotic circumvention (apomeiosis), apospory, parthenogenesis, polyploidy, hybridity and environment (Roy, 1995; Schranz et al., 2005; Rushworth et al., 2011). *Boechera genus* in particular comprises of numerous independently emerged asexual (sub)species indicating that this genus may harbor a specifically pronounced (epi)genetic background favorable for evolution of apomixis. Along with polyploid apomicts, *Boechera* genus also includes diploid apomictic populations that were presumably sired from apomictic polyploids or aneuploids, or occurred via hybridization (Kantama et al., 2007; Lovell et al., 2013). Apomixis in diploid condition is extremely rare, and polyploidy and hybridity seems to have been the springboard for evolution and maintenance of apomixis (Comai, 2005; Hand and Koltunow, 2014; Hojsgaard et al., 2014b). Unlike the polyploid apomictic parents, the rare apomict diploids might display segregation of apomictic traits (Noyes and Wagner, 2014) and fitness trade-offs, hence apomictic diploids do not seem to survive in most apomictic genera. In triploids, where meiotic synapsis of chromosomes fails causing so-called “triploid block,” apomixis remains the only possible mean of reproduction through seeds; to some extent this concerns other polyploids too. However, as most diploid apomicts in *Boechera* seem to represent products of hybridization (Beck et al., 2012), a similar “genomic block” for meiotic bivalent formation may take place. Yet another route for evolution of a triploid apomict might have been endo-duplication due to triggered stress response following exposure to harsh environmental conditions (Scholes and Paige, 2015). This scenario might have led to diploid (sexual) gamete in one genotype, which was then fertilized by a haploid sexual gamete, thus resulting in triploid genotype. We reported similar endo-duplication events in an *Arabidopsis* mutant (Johnston et al., 2010), and we believe it was due to genomic stress in the female gamete mediated by loss of a crucial cell cycle regulator. Especially triploids could have paved way for evolution of apomixis by fixing and maintaining heterozygosity. Spontaneous apospory reported in *Ranunculus* triploids (Hojsgaard et al., 2014a) might lend additional support along this line. In addition, asynchronous gene expression from different genomes is thought to facilitate asexual re-programming in hybrids (Carman, 1997; Hojsgaard et al., 2014a). As hybridity leads to “genomic shock” thus “genomic stress,” we may not be able to tease apart cause and consequence.

Understanding apomixis traits has been mostly restricted to transcriptome analyses of apomixis-specific organs or cell-types, and population genetic studies. For instance, Sharbel et al. (2010) reported heterochronic changes in gene expression during initiation of apomixis along with decrease of their transcript levels in the apomict by comparing expression tags across four stages of ovule development between diploid apomictic and diploid sexual *Boechera* species. Another study analyzed apomictic cell type-specific (e.g., MMC) gene expression profiles of a triploid apomictic *Boechera* similar to the one we used in this work, and compared to the corresponding profiles of a distantly related (10 million years of divergence) sexual species *A. thaliana* (Schmidt et al., 2014). Although number of interesting gene expression changes related to meiotic and mitotic cell cycle regulation, hormonal pathways, signal transduction, stress and epigenetic regulatory pathways were alluded to be specific for the apomeiotic MMC, comparative analyses seem to be constrained due to the lack of appropriate sexual control. It was also noted that all major core meiotic genes were expressed in the apomictic MMC in this triploid apomict, and the authors proposed that diplosporous apomeiosis likely result from modification of the core meiotic and cell division pathway in the MMC. Although some candidate loci underlying apomixis have been isolated or reported, functional analyses of apomixis in *Boechera* are limited primarily owing to the inherent difficulties in plant transformation of these species.

**FIGURE 6:**
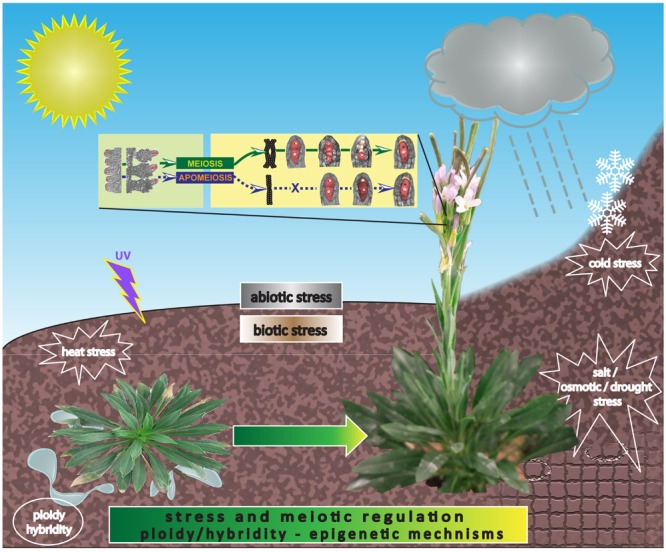
**An artist’s view illustrating how apomictic *Boechera* presumably sired from sexual ancestors, were and are exposed to extreme abiotic and biotic conditions in the natural habitat where they evolved.** Both genetic and epigenetic control of stress and apomeiotic regulation throughout plant development could be viewed as co-adapted gene responses during stress amelioration acting as an efficient buffer to tolerate the mutation load in the absence of meiotic recombination.

Environment has always remained a crucial factor with the highest impact on homeostasis and plant life cycle. Whereas most accessions of the model plant *A. thaliana* are drought-sensitive, the *Boechera* clade in general and a number of *Boechera* species in particular seem to have acquired an asset of biotic and abiotic stress-tolerance mechanisms, perhaps due to their predominant ability to frequently hybridize, forming novel species that occupy distinct ecological niches primarily in their native habitat ranges; and due to their repeated exposure to extreme and fluctuating environmental conditions involving major abiotic stress factors like drought, cold and several biotic factors as depicted in **Figure 6** (Rushworth et al., 2011). Apomixis is absent in the *Arabidopsis* clade but prevalent in the *Boechera*; therefore, it is possible that general stress acclimation could also be an inherent characteristic background for evolution of apomicts, as it might likely provide an efficient (epi)genomic constitution to efficiently tolerate environmental hazards and to buffer mutation loads in the absence of recombination. In parallel, several stress-response genes have been deregulated in transcriptomes of apomictic organs and cell-types of *Boechera* (our unpublished data; Sharbel et al., 2010; Schmidt et al., 2014). In sum, *Boechera* represents an excellent model system for identifying molecular cues to gain in-depth understanding of apomixis regulation and its correlation with environmental change and stress response in a diverse genetic background linked to hybridity and polyploidy.

### Constitutive Stress Response of *Boechera* Apomicts Confers High Abiotic Stress Tolerance

Apomictic *Boechera* in general seem to be more stress tolerant, at least in terms of osmotic stress acclimation, than the corresponding sexual. The apomicts grown in normal conditions exhibit elevated levels of a considerable number of stress-response genes when compared to the sexual (Supplementary Tables S1 and S4, **Table 2**, **Figure 3**). These genes include prominent drought and ABA response factors such as transcription factors NACs, bZIPs, DREB2A, MYBs, and LEA proteins (Nuruzzaman et al., 2013). For example, proper function of bZIP transcription factors AREB1, AREB2 and AREB3 has been shown to be important for drought tolerance (Amir Hossain et al., 2010; Golldack et al., 2011). When overexpressed, *Arabidopsis NAC019, NAC055*, and *NAC072* elevated drought tolerance (Tran et al., 2004).

We noticed though that expression levels of some genes tested were significantly different between the two apomictic accessions used in this study. Both *Apo-1* and *Apo-2* are obligate diplosporous apomicts, and the main difference between them is their evolutionary history including ploidy level (*Apo-1* being triploid and *Apo-2* diploid), ecological habitats with different abiotic stress severity, distant geographical location and, therefore, almost certainly distinct parental genotypes. In order to disentangle the ploidy effect on the gene expression, in the absence of available diploid versus polyploid *Boechera* transcriptome data, we compared our results with published datasets of the *Arabidopsis* clade. About 4% of the *Apo-1-*specific upregulated genes showed the same behavior in *Arabidopsis* allo-tetraploids in an inter-specific hybridization study (Wang et al., 2004), and for stress-related genes it was only 1.8%. Here, three genes *NAC019, NAC055* and *ZML1* from our validated list were upregulated. Similar changes are not discernible in *Arabidopsis* auto-tetraploids compared to the corresponding diploid transcriptomes (Pignatta et al., 2010). Therefore, ploidy might not have been the sole cause of genotype-specific transcription differences and shaped up the entire transcriptome of the *Apo-1*. As *Boechera* apomicts were presumably sexual hybrids by origin that were eventually fixed by apomixis, hybridity between different genomes, thus genome collisions could have played a role for activation of stress-response genes and initiation of apomixis, as evident from upregulation of genes like *NAC019* and *NAC055* homologs in both apomictic floral buds. Upregulation of the homologs of stress-related genes in drought-tolerant apomictic *Boechera* correlates with their crucial function in stress adaptation shown in other plants and support the hypothesis that this constitutive overexpression confers better stress amelioration, in particular osmotic stress tolerance. Our transcriptome data show an intricate network between hormonal regulation and stress response; for instance, preferential activation of several ABA and JA-specific biosynthesis genes and responsive transcription factors in *Apo-1* pinpoints that the apomict has an ability to withstand multiple stresses including osmotic stress and drought (Nguyen et al., 2016). Activation of mobile mRNAs of ascorbic peroxidases (*APX1* and *APX2*) that are pivotal for maintaining cellular redox homeostasis by scavenging hydrogen peroxide and ROS, consequent down-regulation of genes of the auxin biosynthesis and response genes, giving thus a possibility to perturb auxin homeostasis, collectively suggest that the apomict *Apo-1* has an efficient auxin-dependent growth control in order to cope-up with stress in comparison to *Sex-1* (Tognetti et al., 2012; Naser and Shani, 2016), and in turn might activate ABA-specific responses required for stress acclimation. Intriguingly, whereas specific auxin-response factors (ARFs) seem to be down-regulated in seedlings (this study) and many ARFs down-regulated in ovules (Shah et al., unpublished data), they were reported to be enriched in the MMC cells of *Apo-1* (Schmidt et al., 2014), perhaps due to the underlying hormonal homeostasis shifts between the seedling, nucellus stages, and the MMC prior apomeiosis. The latter data, however, is based on comparison between MMC cells of *Arabidopsis* versus *Boechera*, therefore, further validation would be required. The expression of most genes tested showed significant difference between the sexual and apomicts, with ploidy effect being significant at many instances; yet the effect of stress on *in vitro* growth was clearly dependent on the reproductive mode of the *Boechera* species but not the ploidy level. In sum, whether it is *Apo-1*, or the evolutionarily distinct *Apo-2*, there is a clear trend that the *Boechera* apomictic seedlings confer higher level of stress acclimation and underlying gene response in seedlings than the sexual counterpart, and likely play a pivotal role in sustaining apomixis through to apomeiotic transition.

### Does Genome-Wide Deregulation of Meiotic Machinery Underlie Apomixis?

Apomeiotic switch has been perceived as a developmental reprogramming event connected to meiotic shunt, stress, epigenetic and transcriptional changes, transposon control etc. (reviewed in Grimanelli, 2012; Horandl and Hadacek, 2013; Mercier et al., 2015). Previous work in *Arabidopsis* has indicated the importance of meiotic mutations in producing unreduced gametes by apomeiosis-like program. For instance, *DYAD/SWITCH1 (SWI1)* is an important candidate gene in *Arabidopsis* reported for switching the meiosis program toward apomeiosis (Ravi et al., 2008). In *MiMe-1* and *MiMe-2* triple mutants, a diplospory-like division also leads to the formation of unreduced gametes. By combining different meiotic mutant combinations [(*MiMe-1*: *sporulation11-1* (*spo11-1*), *omission of second division1* (*osd1*) and *recombination8* (*rec8*); *MiMe-2*: *spo11-1, osd1* and *cyc1;2/tardy asynchronous meiosis* (*tam*)], diplospory-like meiotic restitution have been successfully recreated in *Arabidopsis* (d’Erfurth et al., 2009, 2010). In maize, a mutation in an effector of small RNA pathway has been shown to be important for recreating diplospory-like event (Singh et al., 2011). Whereas role of these genes or mutations underlying apomixis in a natural apomict like *Boechera* has not yet been documented, *Arabidopsis* versus *Boechera* (*Apo-1*) MMC comparisons suggested possible down-regulation of certain meiotic genes: *PARTING DANCERS, SPO11-2, TAM, SWI1, SOLO DANCERS* and *RAD50* (Schmidt et al., 2014). Whereas this data need real-time validation, none of these genes were deregulated in our transcriptome of *Apo-1* versus *Sex-1* seedlings, but deregulation of two other meiotic genes were noteworthy. Acute down-regulation of core meiotic homologs such as *ASY1* and *MPS1* both in seedlings and apomeiotic buds gives a hint that a global alteration in expression of these genes are likely necessary in order to make the apomeiotic switch. ASY1 is a Horma-domain containing DNA-binding protein component of the meiotic axis involved in synapsis and crossover formation, and it is required for DMC1-mediated interhomolog recombination during female and male meiosis (Armstrong and Jones, 2001; Sanchez-Moran et al., 2007). MPS1 is a coiled-coil protein involved in meiotic spindle formation and it is required for faithful chromosome segregation during female and male meiosis (Jiang et al., 2009). Interestingly, none of the meiotic genes we identified in our study (**Table 1**, Supplementary Table S3) showed transcriptional changes in transcriptomes of *Arabidopsis* polyploids or hybrids (Wang et al., 2004; Yu et al., 2010). Could evolutionary history traits in *Boechera* different from that of *Arabidopsis* influence expression of these genes? It is also likely that this transcriptional homeostasis reflects a genome-wide change in apomictic *Boechera* and might involve heritable epigenetic silencing.

### Meiosis or Apomeiosis: A Stressful Decision Made in Advance?

Abiotic stress has been shown to affect meiotic progression, whereas different type, severity and duration of the stress factors may have different effects on reproduction (reviewed in De Storme and Geelen, 2014). In addition, how stress affects meiosis could be different between male and female tissues. As female tissues are more somatically dependent than the male organs, for instance, female meiosis seems to progress during water deficit but male meiosis abort in *Arabidopsis* (Saini, 1997). In barley, heat stress leads to precocious onset of meiotic chromosome condensation during male meiosis and a concomitant *ASY1* upregulation (Oshino et al., 2007). In *Arabidopsis* seedlings, *ASY1* seems to be marginally down-regulated during drought, and *ASY1* expression levels seem to fluctuate inconsistently between time points during prolonged stress condition (Kilian et al., 2007). The same is true for *MPS1* upon light stress, and for *DYAD* during cold, drought, and heat; therefore, a link between stress response and meiotic machinery may exist much earlier than onset of reproduction itself. However, direct connections between stress response and regulation of meiotic genes in apomicts have not been elaborated earlier. The stress-co-adapted and highly heterozygous apomictic genomes, similarly to polyploids (Chandra and Dubey, 2010; Coate et al., 2013; Hao et al., 2013; Syamaladevi et al., 2016), generally are considered to be a haven for buffering mutation load in the absence of meiotic recombination (Lynch, 1984). Therefore, not only those apomictic plants are well prepared for stress, they also feature “apomeiotic” mutations or down-regulation of genes such as *MPS1* and *ASY1* right from the very growth of the apomictic plant. The broadly adapted apomicts may more efficiently buffer mild abiotic stress than the sexuals, thus limiting cellular oxidative stress and formation of ROS and resulting DNA damage, hence, there is no need for meiosis and recombination in the apomict [sensu. FAR theory (Hadany and Beker, 2003a,b) and oxidative damage initiation hypothesis or meiosis (Bernstein and Bernstein, 2013; Horandl and Hadacek, 2013)]. In agreement with this, in our experimental setting, we observed neither reproducible transcriptional changes of *Boechera* homologs of *MPS1* and *ASY1* in seedlings upon osmotic stress (data not shown) nor reversion of apomeiosis to sexual-like meiosis. Recent preliminary reports indicate that a facultative apomictic *Boechera* species might incline toward sexual meiosis in a substantial fraction of ovules under drought and heat (Carman et al., 2015). These results are also reminiscent of similar observations in a facultative apomict *Ranunculus* under extended photoperiod (Klatt et al., 2016), and collectively hint that residual sexuality contained in the facultative apomicts could be triggered upon environmental cues. In light of our observation that obligate apomictic *Boechera* accessions exposed to mild drought did not exhibit any significant reversion to sexuality in our conditions, we propose that additional stress may not impinge on the reproductive mode of obligate apomicts that were already co-adapted for stress acclimation and meiotic circumvention.

Among several transcription factor groups that are predominantly transcriptionally activated in the stress-tolerant apomict, we have noticed upregulation of the family of NAC transcription factors, which have previously been reported to work in tandem with each other (Hickman et al., 2013). This reproduction mode-specific activation extends beyond seedling to reproductive phase, proposing the prime importance of this regulatory network during apomixis. Stress-response and developmental role of NACs have previously been discussed in various plant systems (Meng et al., 2009; Nuruzzaman et al., 2013; You et al., 2015). It is interesting to note that NAC genes chosen as candidates from our RNA-seq (*NAC019, NAC032, NAC055*) are also known to be expressed in specific cell-types and ovules in *Arabidopsis* (Yu et al., 2005; Johnston et al., 2007; Wuest et al., 2010; Schmidt et al., 2011; Schmid et al., 2012). Although the micro-dissected RNA-seq did not give consistent presence calls for several NACs in laser-captured single cell transcriptomes presumably due to experimental noise, *NAC032, NAC002 and NAC103* homologs of *Boechera* were consistently present in *Apo-1* apomeiotic cell types such as the MMC and surrounding ovular cells (Schmidt et al., 2014) (Supplementary Table S4). A recent work identified novel role of NAC family members in ovule separation (Goncalves et al., 2015), thus in addition to stress regulation, NACs might govern early reproductive development. A number of NACs are also known for their non-cell-autonomous movement via movement of their mobile mRNA (Thieme et al., 2015); therefore, one possibility may be that they maternally control decisions on sexual or apomictic reproductive development.

### Does Epigenetic Regulation Operate in the Apomicts?

Epigenetic regulation involving DNA methylation and histone modifications has long been recognized as a potent regulator of stress-response in plants (reviewed in Probst and Mittelsten Scheid, 2015; Bilichak and Kovalchuk, 2016). Whereas the underlying regulation is extremely complex, a two-step model proposed for transcriptional activation of stress-response genes includes first DNA methylation in CG context, which then directs H3K9 methylation recruiting non-CG methylation (reviewed in Du et al., 2015; Probst and Mittelsten Scheid, 2015). Whereas DNA hypermethylation of stress-inducible genes might lead to enrichment of H3K9me2 and a concomitant depletion of histone acetylation, as shown for *Arabidopsis* seedlings under saline stress (Bilichak et al., 2012), drought activated genes are associated with elevation of H3K4 trimethylation and H3K9 acetylation (Kim et al., 2008). However, epigenetic specificity between DNA and histone methylation under stress might vary between genes, as shown for salt-responsive transcription factors in soybean (Song et al., 2012). A review of the previous data showing locus-specific DNA and histone methylation marks, and small RNAs in *Arabidopsis* (Supplementary Table S4) suggests that the corresponding *Boechera* loci might be under complex epigenetic control. Specifically, *NACs, MYBs*, and several other genes coding for stress-related transcription factors in different plant species have been shown to be targets of miRNAs (Rhoades et al., 2002; Guo et al., 2005), a class of small RNAs involved in post-transcriptional gene silencing and, in many of these cases, induced by stress. In light of these previous findings, our data of *Boechera NAC019* being significantly deregulated in response to global demethylation, supports an underlying epigenetic regulation in the apomicts. The *Arabidopsis NAC019* was previously shown to be activated upon allo-polyploidization in large-scale transcriptomic datasets (Wang et al., 2004), supporting the hybridization effect also in *Boechera*. As *Boechera* apomicts studied here likely represent allopolyploid and diploid hybrids, it is important to consider the importance of epigenetic landscape of the entire genome to be successful apomicts. Global changes in DNA methylation, RdDM-dependent transcriptional changes etc. would be important for the hybrid/polyploid genome to perpetuate apomixis in plant populations (reviewed in Verhoeven and Preite, 2014). Besides, the heterozygous genome and epigenetic landscape instigated by hybridity or polyploidy is compatible with the efficient stress ameliorative advantages in the apomicts, as discussed above.

In addition to the previous data that apomixis is largely epigenetically controlled via pathways of DNA methylation in several apomictic plant systems (Garcia-Aguilar et al., 2010; Zappacosta et al., 2014; reviewed in Hand and Koltunow, 2014), and also by observing the fact that DNA methylation pathway members are deregulated in *Boechera* apomictic cell types (Schmidt et al., 2014) and generally at different developmental stages of *Boechera* apomicts (unpublished data), our data indicating control of *MPS1* and *ASY1* by DNA methylation highlights the importance of an epigenetic angle underlying meiotic deregulation. In this work, we have provided indirect evidence that global DNA methylation might possibly control the apomict-specific transcriptional regulation of stress and meiotic genes in the seedlings in a ploidy-independent manner, though this effect could be coupled to a rather marginal DNA-damaging effect of zebularine (Liu et al., 2015). It is important to note that correlation of DNA methylation status with expression is complex and modifications specific to each cytosine will have to be mapped in the future by locus-specific bisulfite sequencing. Nonetheless, our work provides a base to further investigate the connection between genome-wide and locus-specific DNA methylation and gene expression alterations in *Boechera* species and its possible correlation with the apomixis program. It will also be interesting to examine how epigenetics, the redox status and DNA breaks play a role in regulation of meiotic genes like *ASY1*. Are there connection between apomict-dependent ASY1 regulation, and SPO11 (Horandl and Hadacek, 2013), which are critical for double strand break repair and chromosome pairing, and may their expression be induced by DSB-dependent signaling network at different developmental stages? The decreased levels of *Boechera MPS1* and *ASY1* expression starting from seedling stage along with constitutive stress response in the apomicts allow us to propose that apomictic initiation is pre-programmed from early plant development onward, perhaps due to global (epi)genome-wide deregulation and resulting changes in the transcriptome. Taken together, our work forms one of the first glimpses to view apomixis as a possible read-out of stress-related genome- and transcriptome-wide alterations. Whereas previous studies in *Boechera* have given importance to gene expression changes that occurred during the apomeiotic shift or parthenogenetic reprogramming during the reproductive phase, our work for the first time examines the earliest molecular events during the vegetative phase of the apomicts, and provides a transcriptomic resource for targeted work. The dynamic changes connecting these factors in evolutionary timeline have possibly resulted in genome-wide transcriptome adjustments uncovered in *Boechera*. The work has hinted the importance of the evolutionary history, constitutive stress response elevation, meiotic gene deregulation for priming apomixis in the apomictic species, and we anticipate that this phenomenon will likely be a common background for most, if not all apomicts, similar to the proposals in the apomeiotic dandelion (Verhoeven and van Gurp, 2012). Not only in plants, stress and/or epigenetics are important denominators that impinge on asexual reproductive mode in animals too, for instance in insects and lizards (Kearney et al., 2006; Jedlicka et al., 2015) and in mice (Kono et al., 2004). Nature must have dictated co-evolution of these mechanisms across kingdoms.

## Author Contributions

AJ conceived of the project, and both AJ and JM supervised the wet lab and bioinformatics work, respectively. Lab experiments described here were performed by: JS, OK, PP, and MT; NGS sequencing was performed by Fasteris, Switzerland. Bioinformatic analyses were conducted by JS and JM; annotations, data comparisons, figures and tables were prepared by JS and OK. The manuscript was drafted by JS, OK, and AJ, and all authors read and approved of the content.

## Conflict of Interest Statement

The authors declare that the research was conducted in the absence of any commercial or financial relationships that could be construed as a potential conflict of interest.

## Footnotes

1 www.string-db.org
