# Supplementary material for: Depletion of Key Meiotic Genes and Transcriptome-Wide Abiotic Stress Reprogramming Mark Early Preparatory Events Ahead of Apomeiotic Transition
Source: Front Plant Sci. 2016 Oct 26;7:1539. doi: 10.3389/fpls.2016.01539 (PMC5080521; doi:10.3389/fpls.2016.01539)
Supplement: Supplementary file 6 [file Image_1.PDF]

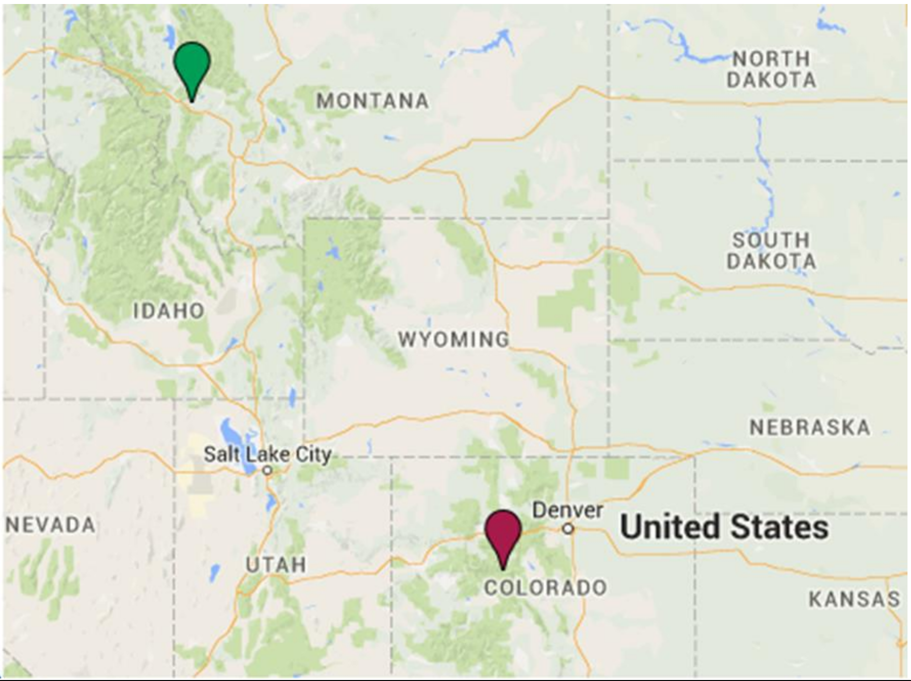

Accession	*Sex-1, Apo-1*	*Apo-2*
Species (ploidy)	*Boechera stricta* (2n=14)<br>*B. gunnisoniana* (2n=3x=21)	*B. divaricarpa* (2n=14)
Site	Gold Creek, Gunnison County, Colorado, USA	Vipond Park, Beaverhead, Montana, USA
Flowering time (months)	6-7 (*Sex-1*), 3-4 (*Apo-1*)	3-4 (*Apo-2*)
Altitude (m)	2530	2263
Latitude (°N)	38°	45°
Longitude (°W)	106°	112°
Temperature mean (°C) in July	26.9	17.2
Temperature mean (°C) in January	-3	-7.2
Rainfall per annum mean (mm)	10.57	31
Reference	Roy, 1990	Schranz et al., 2006

Figure S1. Geographical and environmental particulars of origins of *Boechera* genotypes used in this study. Map source: Google Map
