# Supplementary material for: Depletion of Key Meiotic Genes and Transcriptome-Wide Abiotic Stress Reprogramming Mark Early Preparatory Events Ahead of Apomeiotic Transition
Source: Front Plant Sci. 2016 Oct 26;7:1539. doi: 10.3389/fpls.2016.01539 (PMC5080521; doi:10.3389/fpls.2016.01539)
Supplement: Supplementary file 8 [file Image_3.PDF]

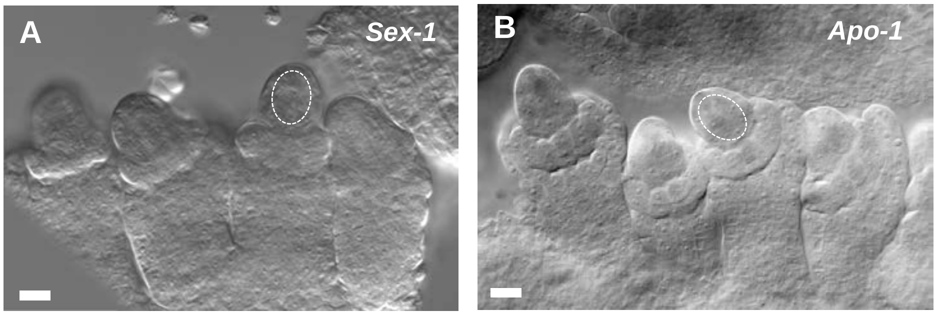

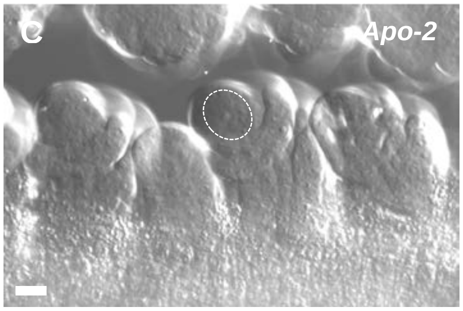

Figure S3. Floral bud staging for qRT-PCR analysis. Bud size for *Sex-1* was 1.6-1.9 mm, for *Apo-1* 1.0-1.2 mm, for *Apo-2* 1.5-1.8mm. The buds contained pre-meiotic ovules of *Sex-1* (**A**), *Apo-1* (**B**) and *Apo-2* (**C**) enclosing Megaspore Mother Cells (MMC, circled). Scale bar 10µm.
