# Supplementary material for: Depletion of Key Meiotic Genes and Transcriptome-Wide Abiotic Stress Reprogramming Mark Early Preparatory Events Ahead of Apomeiotic Transition
Source: Front Plant Sci. 2016 Oct 26;7:1539. doi: 10.3389/fpls.2016.01539 (PMC5080521; doi:10.3389/fpls.2016.01539)
Supplement: Supplementary file 7 [file Image_2.PDF]

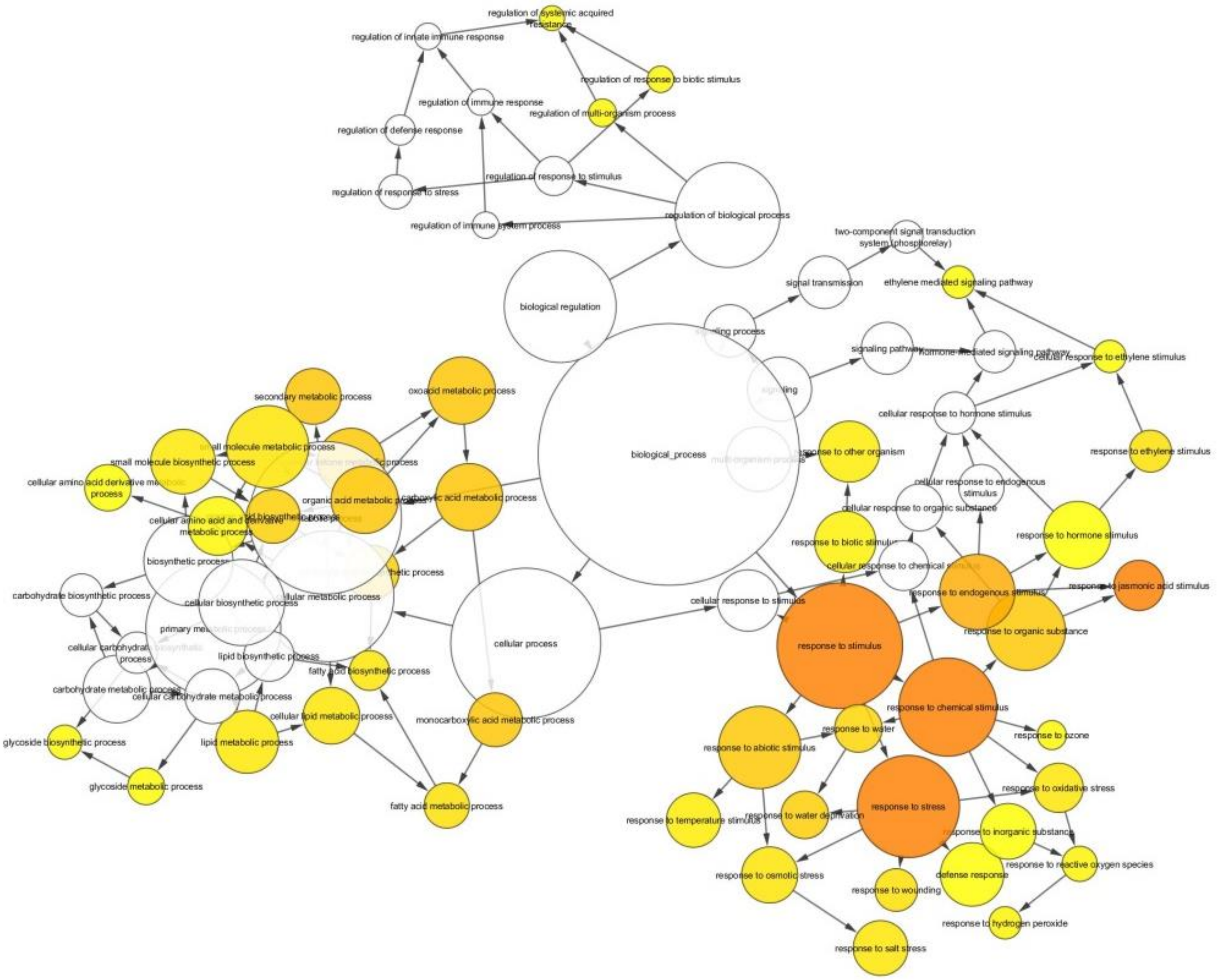

Figure S2. Annotation network of genes upregulated in *Apo-1* by 2 folds. The network was generated using Bingo tool from Cytoscape with Benjamini and Hochberg FDR correction with p-value cut-off of ≤ 0.05.
